# Supplementary material for: Illness-related suffering and need for palliative care in Rohingya refugees and caregivers in Bangladesh: A cross-sectional study
Source: PLoS Med. 2020 Mar 3;17(3):e1003011. doi: 10.1371/journal.pmed.1003011 (PMC7053708; doi:10.1371/journal.pmed.1003011)
Supplement: S1 Text — (DOCX) [file pmed.1003011.s002.docx]

**Proposal**

**Palliative care needs of Rohingya refugees in Cox's Bazar: a needs assessment**

**Background Information & Rationale**

BACKGROUND: Most refugees living in refugee camps struggle to access primary care services and have a very different set of healthcare needs (1). Refugees often arrive in poor health, and mortality risks are frequently highest immediately after reaching their country of asylum, where they are dependent on foreign aid (2). They must endure extreme living conditions, with poor hygiene, a lack of basic supplies including food and clean water, as well as a lack of resources and access to health care. These conditions increase the risk of diseases such as cholera, typhoid and shigella. In addition, crowding and low immunization coverage brings the threat of measles and rubella. Pneumonia and diphtheria are also significant causes of mortality and innumerable children are being treated for severe acute malnutrition. The WHO has called on healthcare professionals to action to provide care and protection to these individuals. However, palliative care has been omitted from relief strategies in refugee camps around the world, despite the 2014 World Health Assembly Resolution 67 requesting palliative care to be integrated into health systems.

A large number of refugees facing chronic or life-threatening illness go unreached by humanitarian health aid; and many of those who could have been provided essential palliative care and pain relief will not receive it before they die. These individuals often require specialized ongoing treatment that is usually unavailable in humanitarian crises, such as in Cox’s Bazar. Humanitarian teams and international agencies must therefore address the needs of these patients, so that no individual should live or die in severe distress.

Palliative care would enable patients and their families facing life-threatening illness, with physical, emotional, social or spiritual distress, to be supported. Relieving the burden of pain and suffering associated with disease and illness must be a priority in this humanitarian crisis.

Little work has been done to identify and address the palliative care needs of people in current complex humanitarian crises who are living with life-limiting illnesses.

RATIONALE: In Cox’s Bazar and other refugee camps, humanitarian aid agencies and local health services are providing food, water, shelter, immunizations and hygiene kits to stop the spread of disease. The main focus is on preventing and treating acute and curative disease. However, thousands of adults and children are facing chronic and/or life-threatening diseases for which they are not receiving the care they need. Those with HIV, cancer, diabetes and other non-communicable diseases remain unseen and their needs are largely if not wholly unmet, leading to pain and suffering for patients and their families. Palliative care in humanitarian crises has been given little focus or research investment. Data from a palliative care needs assessment will allow for a better understanding of who and how many would benefit from palliative care in refugee camps and would build evidence about how to implement effective programs. It would also allow for monitoring of progress towards reducing the burden of preventable pain and suffering in humanitarian crises.

# Study Objectives

To identify and characterize the palliative care needs of the Rohingya refugees in Cox’s Bazar, including patients, caregivers, healthcare facilities and pharmacy’s needs, to better address their needs through humanitarian healthcare agencies and local healthcare workers.

# Eligibility Criteria

All individuals and caregivers with serious health conditions as identified via house to house surveys in the refugee camps. Healthcare facilities and pharmacies in geographic proximity to the Rohingya Refugee Camps with be included.

# Potential Risks & Benefits

Studies suggest that refugees are often afflicted with healthcare conditions requiring long-term specialized care which is difficult to obtain in camp settings. Patients in refugee camps and communities would benefit from receiving palliative care services which are often inaccessible. Identifying their exact palliative care needs would allow for healthcare professionals, humanitarian teams and primary care providers to be trained in pain and symptom management, and in offering psychosocial support. This would also allow healthcare providers to better address emotional, spiritual and psychological conditions to provide some support to this vulnerable population. Therefore, the results of this study will guide healthcare providers as well as Government and Non-Government Organizations to design programs that will better meet the palliative care needs of refugees in camp settings such as Cox’s Bazar. This will allow for the provision of higher quality end-of-life and palliative care to patients suffering from life-threating and/or chronic conditions.

4. Interviews will be conducted in Cox’s Bazar District using the proposed interview guides, we anticipate an iterative process of interview guide review and revision during the training and piloting phase of the study.

a) Standard Caregiver Tool

b) Standard Bereaved Caregiver Tool

c) Standard Patients tool

Interviews with healthcare facilities will consist of questions regarding the palliative care needs of patients which attend these facilities and the availability of essential package medications and supplies.

# References

1. Abi Nader H, Watfa W. Why be a refugee camp doctor: the challenges, rewards and medical education aspects. International Journal of Medical Education. 2017;8:307-308.

2. Marston J, De Lima L, Powell R. Palliative care in complex humanitarian crisis responses. The Lancet. 2015;386(10007):1940.
