# Supplementary material for: Illness-related suffering and need for palliative care in Rohingya refugees and caregivers in Bangladesh: A cross-sectional study
Source: PLoS Med. 2020 Mar 3;17(3):e1003011. doi: 10.1371/journal.pmed.1003011 (PMC7053708; doi:10.1371/journal.pmed.1003011)
Supplement: Attachment — Submitted filename: Response to reviewers.docx [file pmed.1003011.s003.docx]

Response letter

Please find that itemized list of responses to reviewer and editor comments:

| Comments: Editors | Author response | Line Number (Marked up Manuscript) |
| --- | --- | --- |
| Please begin the process of depositing study data in a public repository. | We have deposited into Cambridge University Repository - Apollo  Title: "Palliative care needs in Rohingya refugees 2017" – Placeholder DOI link: <https://doi.org/10.17863/CAM.45776> | - |
| Please revise your title to better match journal style. We suggest "Illness-related suffering and need for palliative care in Rohingya refugees and caregivers in Bangladesh: a cross-sectional study". | Title revised to “Illness-related suffering and need for palliative care in Rohingya refugees and caregivers in Bangladesh: a cross-sectional study". | 1-2 |
| To your abstract, please add the start and end dates of recruitment for your study; and brief demographic details for study participants. | Modified to “Between Nov 20-24, 2017, we conducted a cross-sectional study of individuals with serious health problems (n=156, 53% male) and caregivers (n=155, 69% female) living in Rohingya refugee camps in Bangladesh. The most common diagnoses were significant physical disabilities (n=100, 64.1%), treatment-resistant tuberculosis (TB) (n=32, 20.5%), cancer (n=15, 9.6%), and HIV infection (n=3, 1.9%).” | 63-70 |
| The final sentence of the "methods and findings" subsection of your abstract should summarize the study's main limitations. | Added: ” The main limitations of this study were the use of convenience sampling and close-ended interview questions.” | 71 |
| Around line 67, please begin your discussion of conclusions by referring to your findings (i.e., "In this study, we found that ..." or similar). | We have revised with the suggested phrasing | 73-75 |
| The "Lancet Commission" is mentioned three times in your introduction, and we suggest that once would suffice. | Introduction has been revised to remove 2 of the references. |  |
| After the abstract, we will need to ask you to add a new and accessible "author summary" section in non-identical prose. You may find it helpful to consult one or two recent research papers published in PLOS Medicine to get a sense of the preferred style. | This has been added | 104-148 |
| Early in the methods section, please state whether the study had a protocol or prespecified analysis plan, and if so attach this as a supplementary file (referred to in the text). Please highlight analyses that were not prespecified. | We have clarified that there was no prespecified analysis plan |  |
| Throughout your text, please format reference call-outs as follows: "...saving lives [1,3]." | This has been done (although we cannot figure out how to get square brackets instead of round) |  |
| Please avoid using italics for emphasis. | Italics have been removed | 216 |
| Please substitute "sex" for "gender" as appropriate. | This has been done | Table 1,4  Line |
| Please remove the "Role of the funding source" section from your text. | This has been removed |  |
| In your reference list, please ensure that journal name abbreviations are consistently observed (e.g., "Lancet" for references 2 and 3; "JAMA" for reference 27. | This has been corrected |  |
| Please add full access details to references 2 and 7. | Ref 2- this is corrected  Ref 7-removed as this was not felts to be particularly relevant |  |
| Please add author names where available, e.g. to references 11 and 12; please format the author names for reference 1 consistently with other references; and make the author of reference 4 "WHO" (or the fully spelt-out version). | All references were reviewed and corrected as appropriate |  |
| Please correct the typo in reference 12. | Corrected |  |
| You mention the STROBE reporting guideline in your text, and we ask you to add a completed checklist as a supplementary file, referred to in the methods section. In the checklist, individual items should be referred to by section (e.g., "Methods") and paragraph number rather than by line or page numbers, as the latter generally change in the event of publication. | We have added STROBE as a supplementary file with the requested changes |  |
|  |  |  |

Comments from the reviewers:

Reviewer #1:

| Reference 4 needs to be corrected (World Health Organization) Reference 7 does not seem to be complete | Corrected as mentioned above |  |
| --- | --- | --- |
| Page 7, line 128:  When exactly was the data collection period? | This has been added | 316 |
| Page 7, line 134: how many experts provided feedback? | This has been clarified, numbers added | 244-245 |
| Page 7, line 138: did the authors consider cultural adaptation of the questions and if so, how? | This has been clarified: “This pilot schedule was tested with 10 Rohingya interviewers from the refugee camps and 20 Rohingya individuals (11 with serious illnesses, 9 caregivers) and modifications were made to improve the clarity of questions and response options to reflect the specificities the Rohingya refugees’ experiences and culture.” | 248-250 |
| Page 8, line 156: how many interviewers were trained? Did I understand correctly that there was only one training workshop and an 8-day sampling period immediately following that workshop? When did you have the workshop? What contents were included in the practical and the theoretical parts of the workshop? | We have added the following as clarification:  **“Interviewer Training and Linguistic Adaptation**  Two co-authors (MD, FK), who had previous experience with conducting similar studies in Bangladesh, recruited and trained ten Rohingya –speaking interviewers to conduct structured interviews for this study. The interviewers were identified through a partnership with a local health non-governmental organization working in the refugee camps (OBAT Helpers), who had previous employed the majority of these individuals in various health promotion and/or translator roles for programs in the refugee camps. All interviewers had completed secondary school and were fluent in both written and spoken English and in the Rohingya language (spoken only, as there is no widely accepted written form of Rohingya). All interviewers were of Rohingya ethnicity, and the majority (90%) had been living in the refugee camps for more than 5 years, having arrived during previous waves of refugee movement from Myanmar.  Interviewer training was conducted in English and consisted of two days of theoretical and practical in-person training, which included didactic teaching and practical examples related to research ethics and informed consent, the goals of the study, and key concepts related to the study (palliative care, serious health problems, cancer, HIV/AIDS, medications, medical equipment, and symptoms). During the training, the interviewers reviewed each question in the interview guide with the trainers, discussed the meaning of the question and the response options, and provided suggestions about cultural or other adaptations which would improve the clarity of the interview guide. The interviewers then agreed upon a single translation of each interview question into Rohingya, after group discussion. During the practical portion of the training, interviewers conducted practice interviews in pairs, with observation and coaching by the trainers who provided feedback about interview technique and clarified the interview guide questions and response options. During the final phase of training, interviewers conducted interviews in the refugee camps, under the same conditions as those in the actual study, with observation by the two trainers, to ensure fidelity to the interview guide. The interviews were conducted over a 5-day period (Nov 20-24, 2017) immediately following training. Interviews typically took 30-45 minutes.” | 280-319 |
| Page 10, line 188: are you able to estimate how many patients were approached in the recruitment period? Where did the interviewers identify and approach patients? Did they participate in clinics? | We did not record the number of individuals approached.  We have added the following about individuals were identified and approached: “Individuals were identified at the community level, and interviewers spoke to individuals in the community and went from house to house and inquiring about the presence of seriously ill individuals or caregivers for such individuals. Once identified, these individuals were approached about their interest to learn about the study, if individuals agreed, then the interviewer would explain the study and then ask if the individual would be willing to participate.” | 218-223 |
| Page 13, table 2: antibiotics or vitamins /minerals would not be considered pain relieving medication. Could you include this point in the discussion? | We have clarified which treatments would be considered pain relieving medications  Question for reviewer: We could combine all non-pain relieving treatments if this would improve clarity –would appreciate your feedback on this | Table 2 |
| Page 21, line 333: you should compare the lack of opioid availability and accessability to the INCB data or any other data on opioid consumption in Bangladesh | Added the following:  “Despite morphine and other opioids being included on the Bangladesh Essential Drug List, morphine is not practically available outside of the capital city of Dhaka, and the International Narcotics Control Board reports only 18 kg of morphine was consumed in 2017 (14,19).” | Lines 524-526 |
| Page 21, line 339: do humanitarian organizations such as MSF bring opioids to the Rohingya refugee camps? If not, why not? | This is a difficult question to answer, depends on the country and the situation, but no specific data on this is available. In this setting, clearly very few are accessing these meds even if they’re being imported (we did talk to a number of humanitarian health organizations and only one had opioids available, and only for perioperative use). We found that organizations are not keen to speak openly about this subject.  We have added:  “There are no published studies about the availability of opioids in humanitarian crisis situations and how often humanitarian health organizations are able to import these medications as part of their relief efforts. Humanitarian health organizations may choose not to include opioids in their essential supply packages, fearing that country-specific opioid policies may delay the import of the entire shipment of essential medications and supplies, which will hamper relief efforts, especially during urgent crisis situations.” | 532-538 |
| Page 23, line 379: another limitation that you should discuss here is that you could not quantify SHS. The Lancet report gave a good methodology for quantification of SHS, and I would have loved to see some data for example on days with SHS. | Unfortunately, the Lancet commission was published only a month before our study, so we were not able to incorporate the SHS quantification.  Updated:  “. Additionally, we did not attempt to quantify serious illness-related suffering in our study.”  “Such studies should also incorporate the suggested methodology for quantifying serious illness-related suffering and should evaluate programs designed to alleviate the burden of serious illness-related suffering in humanitarian settings and validate published guides and recommendations, which are primarily based on expert recommendations(2). “ | Line 584  589 |

Reviewer #2

| Other than that, my only concern is the translation into Rohingya. Translation is tricky; translation of questions about pain can be particularly tricky. I remember one study (although I don't remember the citation) of menstrual pain in bilingual Chinese-American women. The questions were carefully translated, then back tranlsated into English, then edited. The questionnaire was given to the women in both languages and there were significantly different results.\ | We have address the translation and the limitations of it in methods section  “During the training, the interviewers reviewed each question in the interview guide with the trainers, discussed the meaning of the question and the response options, and provided suggestions about cultural or other adaptations which would improve the clarity of the interview guide. The interviewers then agreed upon a single translation of each interview question into Rohingya, after group discussion.” | 308-310 |
| --- | --- | --- |

Reviewer 3:

| The term "health-related suffering" is an oxymoron. While it was used in the Report of the Lancet Commission on Palliative Care, this seems misguided. Please use instead  "illness-related suffering." | We have replaced this as suggested | Throughout paper |
| --- | --- | --- |
| Per the WHO document entitled "Integrating palliative care and symptom relief into the response to humanitarian emergencies and crises: a WHO guide." palliative care is not only for people with "life-limiting condition." In humanitarian crises, many people, perhaps most people, who suffering requires palliative care may not have a "life-limiting condition." Please make clear, per WHO guidance, that palliative care should respond to the local need and that this need varies by location, culture, and socio-economic situation. | We have modified the following related to this:  Abstract “There is very limited evidence about the need for palliative care and symptom relief to guide the implementation of programs to alleviate the burden of serious illness-related suffering in these settings.”  “Between November 20-24, 2017, we conducted a cross-sectional study of individuals with serious health problems…”  “In this study we found that many individuals with serious health problems experienced significant physical, emotional and social suffering due to a lack of access to pain and symptom relief and other essential components of palliative care. Humanitarian responses should develop and incorporate palliative care and symptom relief strategies which address the needs of all people with serious illness-related suffering and their caregivers.”  Introduction “In humanitarian settings, the need for palliative care and symptom relief often extend beyond individuals with life-limiting conditions, since suffering may occur for many individuals with acute or non-life threatening conditions because there is less access to therapies to prevent, diagnose or treat disease and social supports, thus palliative care must respond to the specific local needs during a humanitarian crisis (3)(2).” | Line 57  Line 63-64  Line 73-99  Line 160-164 |
| Your study population is not limited to people with "life-limiting conditions." It includes people with "significant physical disability", burns, drug-resistant TB, and HIV infection. At least some of these people may live long lives, and some may lead relatively healthy long lives. Thus, please change the term "life-limiting condition" to "serious health problem." | We have changed life-limiting condition to serious health problem throughout the text |  |
| Line 70: Should state: " ... palliative care which addresses the needs of all people with serious illness-related suffering and their caregivers." | Changed to “Humanitarian responses should develop and incorporate palliative care and symptom relief strategies which address the needs of all people with serious illness-related suffering and their caregivers.” | Lines 75-98 |
| More relevant and up to date citation: Krakauer EL, Daubman BR, Aloudat T. Integrating palliative care and symptom relief into responses to humanitarian crises. Med J Australia 2019;211;201-203. | Change made to reference as suggested | Line 159 |
| Lines 107-108: Text should recommend the essential package of palliative care described by WHO specifically for humanitarian crises, not the general package from the Report of the Lancet Commission. | The WHO package was not published at the time our study was conducted, so we did not have access to this list. |  |
| In light of the documented lack of access to disease treatment, please emphasize that palliative care is never a substitute for disease treatment and that efforts to make palliative care accessible for people affected by humanitarian crises should be accompanied by and integrated with efforts to make disease prevention, early diagnosis and treatment accessible to the same population. | We are not sure how this fits with the focus of our study on assessing illness-related suffering and the need for palliative care |  |
